# Body mass index and persistent pain after breast cancer surgery: findings from the women’s healthy eating and living study and a meta-analysis

**DOI:** 10.18632/oncotarget.17948

**Published:** 2017-05-17

**Authors:** Yuan-Yuan Ding, Peng Yao, Lang Wu, Zhen-Kai Han, Tao Hong, Yong-Qiang Zhu, Hong-Xi Li

**Affiliations:** ^1^ Department of Pain Management, Shengjing Hospital of China Medical University, Shenyang, China; ^2^ Division of Epidemiology, Department of Medicine, Vanderbilt Epidemiology Center, Vanderbilt-Ingram Cancer Center, Vanderbilt University Medical Center, Nashville, Tennessee, USA

**Keywords:** body mass index, persistent pain, breast cancer, prospective study, meta-analysis

## Abstract

The purpose of this study is to evaluate the association between body mass index (BMI) and persistent pain after breast cancer surgery in a prospective study and synthesize available evidence through a meta-analysis. In the Women's Healthy Eating and Living (WHEL) Study, 3,088 women diagnosed of breast cancer were enrolled and assessed. After 4 years, a subgroup of 2,131 women was re-assessed for the pain information. Logistic regression models were used to assess the associations of baseline BMI and BMI change between baseline and 4 years of follow-up with general pain symptoms at 4 years of follow-up. We further synthesized all available evidence from observational studies by searching PubMed and Embase up to February 2017. In the WHEL study, baseline BMI was linearly associated with an increased risk of persistent pain at 4 years of follow-up (odds ratio (OR) (95% confidence interval (CI)): 1.07 (1.05-1.10)). After adjusting for baseline BMI, BMI change since baseline was associated with persistent pain (OR (95% CI) for every unit increase: 1.10 (1.04-1.16)). After searching the literature, additional eight studies were eligible to be included in the meta-analysis. After pooling estimates from all nine studies, there was a positive association with persistent pain development comparing obesity or overweight with normal weight. Available data suggested a linear relationship between BMI and persistent pain (OR (95% CI) for every one unit increment of BMI: 1.04 (1.02-1.07)). Overall, our analyses suggested that BMI might be positively associated with risk of persistent pain after breast cancer surgery.

## INTRODUCTION

Breast cancer remains the most common cancer in females [1, 2]. In US alone, it is expected that there will be approximately 252,710 new breast cancer cases among females in 2017 [1]. The 15-year survival rate for breast cancer patients is approximately 78% [3], however, a large proportion of surviving breast cancer patients who have undergone surgery have persistent pain [4], which greatly affects patients’ quality of life. To identify risk factors for persistent pain after breast cancer surgery is critical for developing strategies to decrease its public health burden. Body mass index (BMI), a modifiable factor, has been suggested to be potentially associated with persistent pain by several epidemiological studies [5–7]. For example, compared with normal weight, obese patients were more likely to develop persistent pain after surgery in two prospective studies conducted in Denmark [6, 7]. Another study conducted in Finland suggested that every unit increase of BMI was significantly associated with an increased risk of persistent pain [5]. However, several other studies did not identify a significant association between BMI and persistent pain after surgery [8–13]. The existing studies often vary extensively regarding the lengths of follow-up period, which are usually not very long. It is critical to better characterize the association in a sufficiently powered prospective study with a long follow-up period.

Considering the inconsistent findings across different studies evaluating the association between BMI and persistent pain after surgery in breast cancer patients, a systematic review and meta-analysis synthesizing available evidence would be important to carefully evaluate findings and level of evidence from each study. A meta-analysis summarizing observational studies up to March 2015 revealed a null association between BMI and persistent pain [4]. However, this meta-analysis only assumed a linear relationship between BMI and persistent pain in the data synthetization. Detailed analyses based on specific categories of BMI, namely, obesity, overweight and normal weight, were not assessed. A non-linear relationship, albeit being possible, was also not evaluated. Furthermore, several additional studies evaluating the association of interest have been published since the publication of the previous meta-analysis [6, 7]. A more comprehensive, up-to-date meta-analysis is thus needed to better understand the research question of interest.

In the current study, we aim to better understand the relationship between BMI and persistent pain in breast cancer patients after surgery by analyzing the Women's Healthy Eating and Living (WHEL) Study, a study with relatively long period of follow-up, and synthesizing all available evidence from observational studies through a comprehensive meta-analysis. Findings from such a study may help determine whether BMI, a modifiable factor, could be one of the strategies to decrease the possibility of persistent pain, a common burden for the most frequent malignancy in females of most countries.

## RESULTS

### The WHEL study

The detailed information for the analyzed subsample at 4 years of follow-up was described previously [11]. Compared with subjects recurred or died or did not answer the questionnaire, those analyzed tended to have different patterns of age, BMI, ethnicity composition, education, marital status, breast cancer stage, surgery type, chemotherapy, baseline tamoxifen use, and depression. Overall, among the 2,066 subjects with pain information collected at 4 years of follow-up, 1,664 were with at least mild level of pain (80.5%). Logistic regression analyses adjusting for age at diagnosis, radiation, and baseline pain revealed that compared with normal weight (BMI: 18.5-25), both obesity (BMI≥30) and overweight (BMI: 25-30) were significantly associated with an increased risk of developing persistent pain at 4 years of follow-up (odds ratio (OR) (95% confidence interval (CI)): 2.51 (1.79-3.58) and 1.42 (1.08-1.87), respectively; Table 1). We did not adjust for other factors because that no other variables were suggested to be significantly associated with persistent pain based on a previous systematic review and meta-analysis [4]. We detected a linear relationship between baseline BMI and persistent pain with an OR (95% CI) of 1.07 (1.05-1.10). After adjusting for baseline BMI, a BMI change between baseline and 4 years of follow-up was significantly associated with persistent pain (OR (95% CI):1.10 (1.04-1.16)), suggesting an independent effect beyond baseline BMI.

**Table 1:** Characteristics of studies evaluating BMI and persistent pain after surgery in breast cancer patients

Author,publication year, location	Study type	Cases/subject or control (age), duration of follow-up	Categoriesof exposure/reference	OR (95% CI)	Matched/adjustedvariables
**WHEL Study**	CS	1664/2066 (26-70), 4 years	BMI:18.5-2525-30≥30BMI:Every unit increaseBMI change:Every unit increaseBMI:<30≥30BMI:<25≥25	OR1.0 (Ref)1.42 (1.08-1.87)2.51 (1.79-3.58)OR1.07 (1.05-1.10)OR1.10 (1.04-1.16)OR1.0 (Ref)2.22 (1.61-3.12)OR1.0 (Ref)1.80 (1.42-2.29)	radiation, age at diagnosis, baseline pain, baseline BMI
**Additional studies through literature search**					
Alves Nogueira Fabro, 2012, Brazil, Rio de Janeiro	CS	88/168 (mean 58), 7.5 months	BMI:<30≥30	OR1.0 (Ref)0.87 (0.45-1.66)	N/A
Lundstedt, 2012, Sweden	CS	116/873 (28-73), 3-17 years	BMI:<30≥30BMI:<25≥25	OR1.0 (Ref)0.93 (0.54-1.62)OR1.0 (Ref)1.06 (0.71-1.57)	N/A
Shahbazi, 2015, Iran	HC-CS	61/61 (mean 46-48)	BMI:<30≥30BMI:<25≥25BMI:18.5-2525-30≥30	OR1.0 (Ref)0.89 (0.35-2.28)OR1.0 (Ref)0.93 (0.44-1.96)OR1.0 (Ref)0.74 (0.298-1.836)0.748 (0.228-2.459)	education, drug intake, infection, pain before surgery, type of breast cancer, stage of cancer, type of surgery, and adjuvant therapy
Johannsen, 2015, Denmark (nationwide)	CS	614/1872 (18-70), 15 months	BMI:<30≥30BMI:<25≥25BMI:18.5-2525-30≥3030-35≥35	OR1.0 (Ref)1.44 (1.04-2.01)OR1.0 (Ref)1.16 (0.93-1.46)OR1.0 (Ref)0.97 (0.73-1.29)1.46 (1.04-2.06)1.65 (1.14-2.41)0.92 (0.44-1.93)	age, pain at 15 months
Juhl, 2016, Denmark, Aarhus	CS	100/261 (mean 64), 3 years	BMI:<30≥30BMI:<25≥25BMI:18.5-2525-30≥30	OR1.0 (Ref)1.88 (0.99-3.60)OR1.0 (Ref)1.84 (1.10-3.05)OR1.0 (Ref)1.69 (0.92-3.10)2.13 (1.06-4.27)	radiotherapy, axillary procedure
Lash, 2000, US, Boston	CS	91/244 (55+), 16 months	BMI:<30≥30BMI:<25≥25	OR1.0 (Ref)1.40 (0.74-2.66)OR1.0 (Ref)0.94 (0.56-1.58)	N/A
De Oliveira, 2014, Chicago	CS	110/300 (mean 53 or 61), 6 months	BMI:Every unit increase	OR1.02 (0.98-1.06)	age, axillary lymph node dissection, radiation
Meretoja, 2014, Finland	CS	563/860 (mean 57), 12 months	BMI:Every unit increase	OR1.04 (1.01-1.08)	chronic preoperative pain, preoperative hormone therapy, axillary operation, lymphovascular invasion in the primary tumor, class of risk, chemotherapy, radiotherapy, worst preoperative pain in the area to be operated, No. of previous operations, histological size of the primary tumor, No. of metastatic lymph nodes, Beck Depression Inventory score, Spielberger Anxiety Questionnaire

BMI: body mass index; OR: odds ratio; CI: confidence interval; CS: cohort study; Ref: reference; N/A: not available; HC-CS: hospital-based case-control study.

### Meta-analysis

#### Literature search and study characteristics

**Figure 1:**
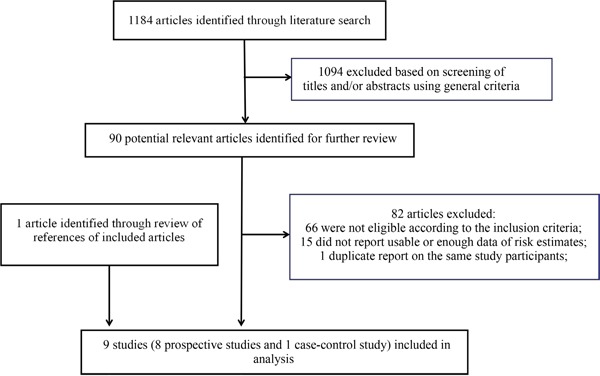
Flow chart for selection of eligible studies

The detailed steps of the literature search and article screening were shown in Figure 1. Including the WHEL study, a total of 9 studies met the inclusion criteria and were included in the current meta-analysis [5–13]. For three studies included in a previous meta-analysis [14–16], there was insufficient information provided in the original publications. We contacted authors of these studies but did not receive necessary information. These studies were thus not included in the current study. The detailed characteristics of the included studies were shown in Table 1. Overall, eight prospective studies and a case-control study were available. These studies enrolled 6,766 breast cancer patients with surgery treatment and had a median follow-up of 1.3 years (range 6 months-17 years). Among them, seven of the eight prospective studies (87.5%) and the one case-control study (100%) were with an overall score of ≥7 points and thus were categorized as high quality studies (Table 2).

**Table 2:** Quality assessment of included prospective and case-control studies

Prospective Studies									
Study	Exposed cohort represents average in community	Selection of the non-exposed cohort from same community	Ascertain exposure through records or structured interviews	Demonstrate that outcome not present at study start	Exposed and non-exposed matched and/or adjusted by factors	Ascertain outcome via independent blind assessment or record linkage	Follow-up long enough for outcome to occur	Loss to follow-up<20%	Overall Score
Alves Nogueira Fabro, 2012	1	1	1	1	0	1	1	1	7
Lundstedt, 2012	1	1	0	0	0	0	1	1	4
Rief, 2011	1	1	1	0	2	0	1	1	7
Johannsen, 2015	1	1	1	0	2	0	1	1	7
Juhl, 2016	1	1	1	0	2	0	1	1	7
Lash, 2000	1	1	1	1	0	1	1	1	7
De Oliveira, 2014	1	1	1	1	2	0	1	1	8
Meretoja, 2014	1	1	0	1	2	0	1	1	7
**Case-Control Studies**									
**Study**	**Case defined with independent validation**	**Representativeness of the cases**	**Selection of controls from community**	**Statement that controls have no history of outcome**	**Cases and controls matched and/or adjusted by factors**	**Ascertain exposure by blinded structured interview**	**Same method of ascertainment for cases and controls**	**Same response rate for both groups**	**Overall Score**
Shahbazi, 2015	0	1	1	0	2	1	1	1	7

1 means study adequately fulfilled a quality criterion (2 for case-control or exposed-non exposed fully matched and adjusted), 0 means it did not. Quality scale does not imply that items are of equal relevant importance.

#### Obesity or overweight versus normal weight

**Figure 2:**
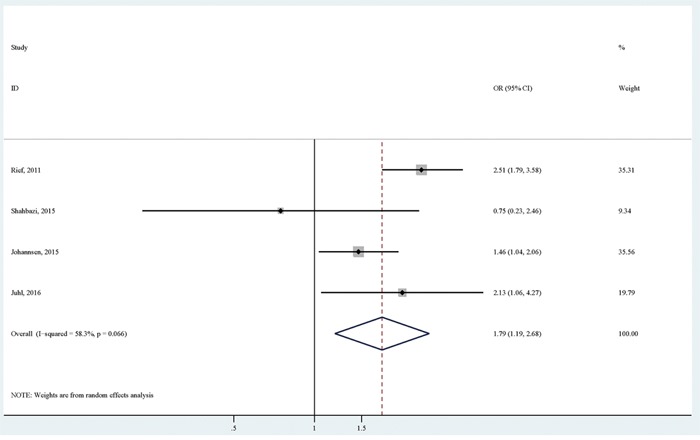
The association between obesity versus normal weight and persistent pain in breast cancer patients after surgery

Four studies reported the association of BMI with persistent pain using normal weight as the reference group. Focusing on obesity versus normal weight, the pooled analysis of available studies [6, 7, 10, 11] revealed a significantly positive association (OR (95% CI):1.79 (1.19-2.68)), with relatively considerable heterogeneity (I^2^=58.3%; *p* for heterogeneity: 0.066; Table 3; Figure 2). There was no significant publication bias as indicated by Egger's test (*p* for bias: 0.609) and Begg's test (*p* for bias: 1.000). Sensitivity analysis revealed that the four study-specific ORs (95% CIs) ranged from as low as 1.50 (1.11-2.02) (I^2^=13.5%) after omitting the study by Rief et al to as high as 2.25 (1.67-3.04) (I^2^=46.0%) after omitting the study by Johannsen et al.

**Table 3:** Summary risk estimates of the association between BMI and persistent pain after breast cancer surgery

	No of reports	OR (95% CI)	I^2^ (%)	P for heterogeneity
**Obesity vs normal (BMI: ≥30 vs 18.5-25)**	4	1.79 (1.19-2.68)	58.3	0.066
**Overweight vs normal (BMI: 25-30 vs 18.5-25)**	4	1.20 (1.00-1.44)	49.1	0.117
**Obesity vs non-obesity (BMI: ≥30 vs <30)**	7	1.39 (1.04-1.86)	52.9	0.047
**Obesity or overweight vs normal or underweight (BMI: ≥25 vs <25)**	6	1.29 (1.00-1.65)	61.5	0.024
**BMI: per unit increase**	6	1.04 (1.02-1.07)	54.2	0.053

**Figure 3:**
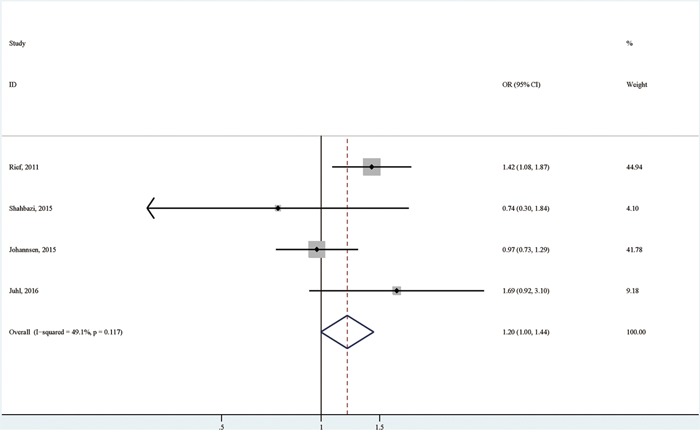
The association between overweight versus normal weight and persistent pain in breast cancer patients after surgery

Compared with breast cancer patients of normal weight, the overweight patients were associated with an increased possibility of having persistent pain (OR (95% CI):1.20 (1.00-1.44); I^2^=49.1%; *p* for heterogeneity: 0.117; Table 3; Figure 3), with no significant publication bias detected by both Egger's test (*p* for bias: 0.916) and Begg's test (*p* for bias: 0.734) [6, 7, 10, 11].

#### Obesity versus non-obesity and overweight or obesity versus normal or underweight

**Figure 4:**
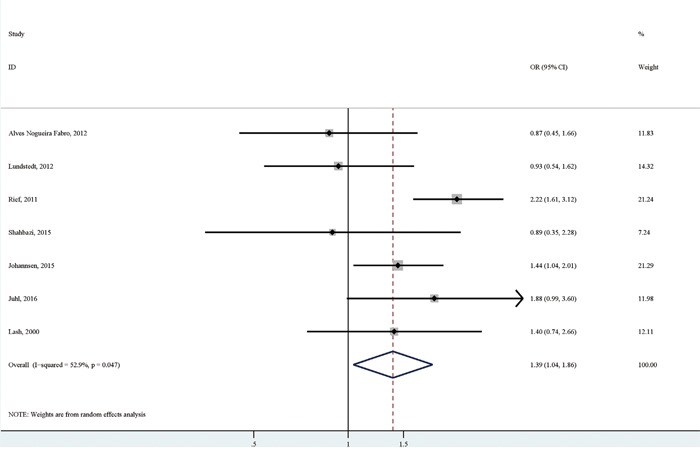
The association between obesity versus non-obesity and persistent pain in breast cancer patients after surgery

Seven studies reported the association of obesity versus non-obesity with persistent pain. The pooled analysis of these studies [6, 7, 9–13] suggested a significantly positive association between obesity and persistent pain (OR (95% CI):1.39 (1.04-1.86)), with relatively high heterogeneity (I^2^=52.9%; *p* for heterogeneity: 0.047; Table 3; Figure 4). There was no significant publication bias as indicated by Egger's test (*p* for bias: 0.149) and Begg's test (*p* for bias: 0.764). Sensitivity analysis revealed that the seven study-specific ORs (95% CIs) ranged from as low as 1.27 (1.02-1.58) (I^2^=2.8%; *p* for heterogeneity: 0.398) after omitting the study by Rief et al to as high as 1.50 (1.12-2.01) (I^2^=46.9%; *p* for heterogeneity: 0.094) after omitting the study by Lundstedt et al.

**Figure 5:**
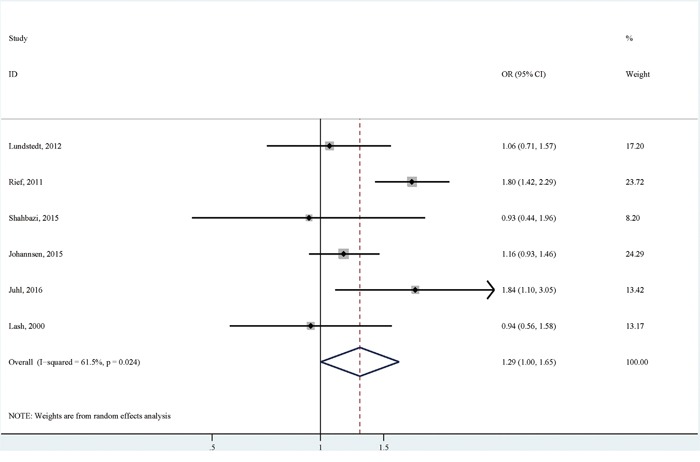
The association between overweight or obesity versus normal or underweight and persistent pain in breast cancer patients after surgery

There were six studies reporting the association of BMI with persistent pain comparing BMI≥25 with BMI<25 [6, 7, 9–12]. Compared with breast cancer patients with no persistent pain, those with persistent pain were more likely to have a BMI≥25 (OR (95% CI):1.29(1.00-1.65), with high heterogeneity (I^2^=61.5; *p* for heterogeneity: 0.024; Table 3; Figure 5)). No significant publication bias was detected by both Egger's test (*p* for bias: 0.532) and Begg's test (*p* for bias: 1.000). Sensitivity analysis revealed that the six study-specific ORs (95% CIs) ranged from as low as 1.16 (0.98-1.37) (I^2^=7.2%; *p* for heterogeneity: 0.366) after omitting the study by Rief et al to as high as 1.32 (0.96-1.81) (I^2^=61.6%; *p* for heterogeneity: 0.034) after omitting the study by Johannsen et al.

#### Dose-response analysis

Three studies provided sufficient data for evaluating potential dose-response relationship between BMI and persistent pain after breast cancer surgery [6, 7, 10]. Additionally, three other studies provided the association of BMI per each unit increase, thus could be included for assessing the linear relationship. Firstly we tested a potential non-linear relationship using data from the three available studies [6, 7, 10]. The likelihood ratio test suggested that there was no sufficient evidence to reject the linear relationship model (*p*=0.10). Based on this, for each of these three studies [6, 7, 10], we generated the association estimate of BMI per each unit increase with persistent pain after breast cancer surgery. We then pooled all association estimates including those from the three additional studies [5, 8, 11]. Overall, we detected a linear relationship between BMI and persistent pain. The overall OR (95% CI) for every unit increment of BMI was 1.04 (1.02-1.07) with persistent pain, with relatively high heterogeneity (I^2^=54.2%; *p* for heterogeneity: 0.053). It seemed that study design, location, follow-up length (≥3 years or <3 years), and case numbers (≥500 or <500) could not fully explain the detected heterogeneity (data not shown). According to the Galbraith plot, the study of Rief, 2011 contributed significantly to the heterogeneity. After excluding this study from the analysis, the overall effect size for the dose-response analysis remained similar (OR=1.03, 95% CI 1.01-1.05), with only minor heterogeneity (*p* for heterogeneity: 0.379).

## DISCUSSION

We analyzed a large prospective study and conducted a comprehensive meta-analysis to assess the relationship between BMI and persistent pain in breast cancer patients after surgery. Based on evidence from the WHEL study and other published observational studies, it seemed that BMI was positively associated with persistent pain, with a linear relationship. These findings demonstrated that decreasing BMI might be one strategy to decrease the possibility of developing persistent pain after surgery for breast cancer patients, if they are replicated and validated by further studies.

Besides persistent pain in breast cancer patients, obesity has been identified to be an independent risk factor for chronic pain after several other surgeries [17, 18]. One potential explanation for the link is that obese females may require more extensive surgery. However, in a study in which most of the subjects underwent a specific surgery - total mastectomy, a higher body weight was still identified to be significantly associated with persistent pain [19]. Also, the previous meta-analysis by Wang et al did not identify an association between breast surgery type and persistent pain [4]. Aligned with our findings of the WHEL study that a further decrease of BMI after the baseline time point might be associated with a decreased risk of having persistent pain independent of baseline BMI, surgery solely might not adequately explains the identified associations of interest.

Our study has several strengths. We assessed the relationship of interest using data from the WHEL study, a large prospective study with relatively long period of follow-up. We were able to adjust for several covariates known to be associated with persistent pain in our analyses, decreasing the possibility of residual confounding. Besides evaluating the associations of baseline BMI by analyzing it as both a categorical variable and a continuous variable, we further assessed the association of BMI change after baseline, which is a unique strength of our analyses. We further performed a comprehensive meta-analysis synthesizing all available evidence, including carefully evaluating potential linear and non-linear relationship of the associations. Our study adds new knowledge for better understanding the relationship between BMI and persistent pain, and provides evidence for supporting the decrease of BMI for decreasing health burden from postoperative persistent pain, if the findings of the current study could be replicated by future studies.

Several potential limitations need to be acknowledged for an appropriate interpretation of our findings. First, in the WHEL study, patients were enrolled after a varying time since the diagnosis of breast cancer, raising the possibility that the baseline BMI and pain information collected and adjusted for in the current analyses may not be exactly the same with those right after cancer diagnosis. However, based on the sensitivity analyses of our meta-analysis including both WHEL and other studies, excluding results from any single study did not significantly revoke the identified positive association between BMI and persistent pain. Secondly, for our meta-analysis, although a large proportion of included studies provided adjusted estimates considering important confounders, residual confounding may still be an issue for biasing the results to some extent. Additionally, for the included studies, there are relatively large differences in the definitions of persistent pain. For example, in the study by Alves Nogueira Fabro et al, pain syndrome information after 6 months of surgical treatment was collected; in the study by Lundstedt et al, reported breast pain at least every week was used to define persistent pain; in the study by Shahbazi et al, post-mastectomy pain syndrome information based on three standard criteria was collected; in the study by Johannsen et al, a pain frequency of almost every day or more frequently was used to define high level of pain; and in the study by Juhl et al, experiencing persistent pain and reporting a nonzero pain intensity in any of the five predefined areas were classified as having persistent pain. In the WHEL study, at least mild level of pain collected at 4 years of follow up was used to determine persistent pain, however we would like to acknowledge that such a definition may not be completely equal to actual persistent pain since the information collected was for the past 4 weeks. As mentioned above, excluding results from any single included studies in our meta-analysis did not significantly influence the identified positive association between BMI and persistent pain. Further large scale well designed studies are warranted to validate our findings. Thirdly, the identified associations from evidence of observational studies cannot infer causality. Additional studies, such as Mendelian randomization studies assessing the association of genetically predicted BMI using genetic variants as instruments with persistent pain in breast cancer patients after surgery are warranted to better understand the causality of the association of interest.

In conclusion, based on evidence from a large prospective study and published observational studies, BMI was positively associated with persistent pain after surgery in breast cancer patients. If replicated in further large-scale well designed studies, our findings may have important implication for decreasing health burden from persistent pain in breast cancer patients.

## MATERIALS AND METHODS

### Women's healthy eating and living (WHEL) study

#### Subjects

The current analysis is based on the WHEL study which includes 3,088 women treated for early stage breast cancer from 7 sites in California, Oregon, Arizona, and Texas. This study was approved by the institutional review boards of each participating institution and the detailed information for this study has been published previously [20–22]. Briefly, the study randomly assigned 3,088 early-stage invasive breast cancer patients (within 4 years of diagnosis) to an intensive diet intervention or to a comparison group between 1995 and 2000 and followed them through 2006. Due to that diet was unrelated to pain, patients were analyzed regardless of their diet assignments in the current analyses.

#### Measurements

Basic sociodemographic variables were collected at baseline by a telephone screening interview and intake forms. Medical records were reviewed to collect clinical data and treatment characteristics. Height and weight were measured during the clinic visits at baseline and at 4 years of follow-up, which were used to calculate body mass index (BMI) at both time points. BMI was categorized into obesity, overweight, normal weight and underweight categories according to the international classifications from the World Health Organization (WHO), which correspond to ≥30, 25-30, 18.5-25, and <18.5, respectively. To assess the level of pain, a composite pain index covering seven pain areas, and items originating from the Symptom Inventory were used, as previously described [11]. Pain items of the Symptom Inventory, which was developed for middle-aged healthy women, were summed up to a general pain score. These were scored between 0 (did not occur), 1 (mild level of pain), 2 (moderate level of pain), and 3 (severe level of pain) for the past 4 weeks. The following pain symptoms were collected: general aches or pains, low back pain, neck pain, headaches or migraines, joint pain or stiffness, belly pain or stomach discomfort, pain or burning while urinating. A reliability index (Cronbach's α=.70) confirms the internal consistency of the composite pain index. In the current analyses, pain was further categorized into two groups, one with a score of 0, representing with less than mild level of pain; and the other with scores of 1, 2 or 3, representing with at least mild level of pain. Pain at both baseline and 4 years of follow-up were collected and used in our analyses. Pain at 4 years of follow-up was used to indicate the persistent pain in analyses of the WHEL study.

#### Statistical analyses

The associations of BMI according to categories of obesity, overweight and normal weight were assessed using logistic regression, adjusting for several known persistent pain risk factors age at diagnosis, radiation and baseline pain [4]. Other factors were not adjusted for because that they were not suggested to be significantly associated with persistent pain based on a previous systematic review and meta-analysis [4]. Besides evaluating the association of obesity versus normal weight and overweight versus normal weight, we also assessed whether BMI≥30 and BMI≥25 are significantly associated with persistent pain compared with BMI<30 and BMI<25, respectively. A potential linear relationship between baseline BMI and persistent pain at 4 years of follow-up was further investigated.

Besides baseline BMI, we further evaluated whether BMI change from baseline to 4 years of follow-up represents an independent risk factor for persistent pain in breast cancer patients. Besides the three potential covariates mentioned above, for this analysis we also adjusted for baseline BMI in the logistic regression model. Analyses for data from the WHEL study were performed using R (version: 3.2.3).

### Meta-analysis

#### Data sources and search strategies

A comprehensive search of PubMed (MEDLINE) and Embase databases was conducted from each database's inception to February 2017, without any language restriction. We used the following search keywords: (“breast cancer” OR “breast neoplasms”) AND (“obesity” OR “overweight” OR “weight gain” OR “weight loss” OR “body weight” OR “weight change” OR “body mass index” OR “body fat percentage” OR “waist circumference”) AND pain. We also screened references of included studies and previous meta-analyses to identify other potential studies.

#### Study selection

Studies were eligible if they (i) were prospective studies or case–control studies; (ii) evaluated the association between BMI and persistent pain after breast cancer surgery; (iii) presented relative risk (RR), odds ratio (OR), hazard ratio (HR) estimates with 95% confidence intervals (CI) or necessary data for determination. There was no restriction for sample size or follow-up duration. If there were several publications from the same study, we included the study with the most cases and relevant information. When there was insufficient information from the study publication or abstract, study authors were contacted to request relevant information.

#### Data extraction and quality assessment

A pair of investigators independently carried out the abstract screening, full text screening, and data extraction. Disagreements were resolved by discussion, with input from other investigators. Data extracted from each study included: the first author's last name, year of publication, study location, study design, characteristics of study population (sample size, age, length of follow-up), and effect sizes of the associations. If multiple estimates of the association for the same outcome were reported, we used the estimate that was adjusted for the most appropriate covariates. In situations when only unadjusted estimates were given, we used the crude estimate.

The qualities of included studies were assessed using the Newcastle-Ottawa Quality Assessment Scale [23]. Specifically, these aspects were assessed: population and sample methods; exposure and outcome descriptions; and statistical matching/adjustments of the data. Each study was then assigned a score (maximum score: 9 points). Studies with an overall score of ≥7 points were categorized as high-quality studies; otherwise they were categorized as low-quality studies.

#### Statistical methods

We used ORs to represent measures of studies evaluating associations of BMI with persistent pain after breast cancer surgery. Same with the WHEL study, the WHO classifications were used to categorize BMI into obesity, over weight, normal weight and underweight groups throughout all included studies. Considering that in our meta-analyses only a limited number of studies were included {von Hippel, 2015 #270}, besides I^2^, we also calculated the *p* for heterogeneity to evaluate the heterogeneity across studies. A *p* for heterogeneity<0.10 was used to determine high heterogeneity [24]. We pooled the log transformed ORs or RRs using the fixed-effects model [25] if there was no obvious heterogeneity. If there was considerable heterogeneity, the random-effects model was used [26]. Sensitivity analyses excluding 1 study at a time were also performed to evaluate whether any specific study significantly influenced the overall pooled results.

For the dose-response analyses, we explored potential non-linear and linear relationship between BMI and persistent pain after breast cancer surgery, using principles as previously published [27]. For studies reporting BMI by categories, we used the midpoint of each category to represent the exposure. If the highest category did not provide the upper bound, we assumed the open ended interval's width to be as same as the adjacent interval's width. We examined a potential nonlinear dose-response relationship between BMI and persistent pain with fractional polynomial models, using restricted cubic splines with 3 knots at fixed percentiles (10%, 50% and 90%) of the distribution [28, 29]. A likelihood ratio test was performed to determine whether nonlinear or linear relationship was suggested.

Publication bias was evaluated via Egger's test [30] and Begg's test [31]. A significance level of 0.05 was used to determine whether there was significant publication bias. Statistical analyses for meta-analyses were performed with Stata (version 13; StataCorp, College Station, TX).
